# Complete genome sequence of the sulfur-oxidizing chemolithoautotrophic *Sulfurovum lithotrophicum* 42BKT^T^

**DOI:** 10.1186/s40793-017-0265-z

**Published:** 2017-09-06

**Authors:** Wooyoung Jeon, Lia Priscilla, Gyuyeon Park, Heeseok Lee, Narae Lee, Dongyup Lee, Hyuksung Kwon, Iksung Ahn, Changha Lee, Hongweon Lee, Jungoh Ahn

**Affiliations:** 1 Biotechnology Process Engineering Center, KRIBB, 40 Yeongudanji-ro, Cheongju, 363-883 South Korea; 2 Bioprocess Department, University of Science and Technology, 217 Gajeong-ro Yuseong-gu, Daejeon, South Korea; 3 Chemical Engineering Study Program, Faculty of Industrial Technology, Institut Teknologi Bandung, Jl.Ganesa No. 10, Bandung, 40132 Indonesia; 4 Department of Chemical and Biomolecular Engineering, National University of Singapore, 4 Engineering Drive 4, Singapore, 117576 Singapore; 5 Department of Chemical and Biomolecular Engineering, Yonsei University, 50 Yonsei-ro, Seodaemun-gu, Seoul, 120-749 South Korea

**Keywords:** Complete genome, Sulfur-oxidizing bacterium, Chemolithoautotroph, CO_2_ bio-mitigation, *Sulfurovum lithotrophicum*

## Abstract

A sulfur-oxidizing chemolithoautotrophic bacterium, *Sulfurovum lithotrophicum* 42BKT^T^, isolated from hydrothermal sediments in Okinawa, Japan, has been used industrially for CO_2_ bio-mitigation owing to its ability to convert CO_2_ into C_5_H_8_NO_4_^−^ at a high rate of specific mitigation (0.42 g CO_2_/cell/h). The genome of *S. lithotrophicum* 42BKT^T^ comprised of a single chromosome of 2217,891 bp with 2217 genes, including 2146 protein-coding genes and 54 RNA genes. Here, we present its complete genome-sequence information, including information about the genes encoding enzymes involved in CO_2_ fixation and sulfur oxidation.

## Introduction

10.1601/nm.3783 are well-known chemolithoautotrophic bacteria found in deep-sea hydrothermal fields that play significant roles in sulfur, nitrogen, and hydrogen flux [1, 2].

10.1601/nm.8874 42BKT^T^ is a sulfur-oxidizing member of 10.1601/nm.3783 that was isolated from deep-sea hydrothermal sediments in Okinawa, Japan [3]. Strain 42BKT^T^ is a Gram-negative, non-motile, and coccoid-to-short-rod-shaped bacterium that utilizes CO_2_ as a carbon source, S or S_2_O_3_^2−^ as electron donors, and O_2_ and NO_3_^−^ as electron acceptors [3, 4]. Recent studies have focused on its potential industrial applications for CO_2_ bio-mitigation, reporting that this strain could convert CO_2_ into C_5_H_8_NO_4_^−^ at a high specific mitigation rate of ~0.42 g CO_2_/cell/h [4].

The CO_2_-bio-mitigation ability of 10.1601/nm.8874 can be improved and optimized through genetic engineering; however, the present lack of genetic knowledge of 10.1601/nm.8874 renders the genetic engineering of this strain difficult. Here, we presented a preliminary description and the general features of 10.1601/nm.8874 42BKT^T^, along with its genome-sequence annotations and interactions with other 10.1601/nm.8873 species. This information would be helpful for improving the use of chemolithoautotrophic bacteria, including 10.1601/nm.8873 species, in industrial applications in CO_2_ bio-mitigation.

## Organism information

### Classification and features

**Fig. 1:**
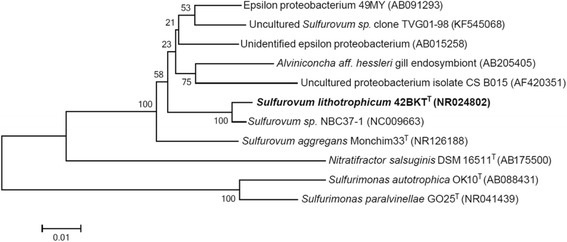
Phylogenetic tree showing the relative position of *Sulfurovum lithotrophicum* 42BKT^T^, based 16S rRNA gene sequence. All sites were informative and free of gaps. Evolutionary history was inferred using the neighbor-joining method [35]. The tree was built using the maximum composite-likelihood method [36]. The percentage of replicate trees with the associated taxa clustered together in the bootstrap test (1000 replicates) is shown next to the corresponding branches [37]. Evolutionary analyses were conducted in MEGA6 [38]. Corresponding GenBank accession numbers are shown in brackets next to the strain name

A representative 16S rRNA gene of 10.1601/nm.8874 42BKT^T^ was compared with that of other species using NCBI BLAST [5]. Figure 1 shows the phylogenetic tree with 10.1601/nm.8874 42BKT^T^, constructed based on the 16S rRNA sequence. This strain shared 99.1% (1393/1406 bp) and 95.1% (1312/1379) sequence identity with the 16S rRNA genes of 10.1601/nm.8873 sp. NBC37–1 [6] and 10.1601/nm.25804 Monchim33^T^, respectively.

**Fig. 2:**
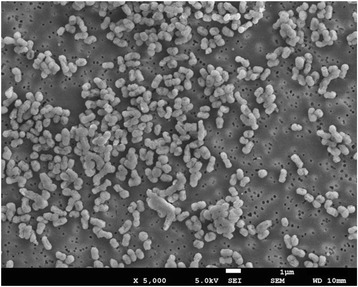
Scanning electron micrograph of *Sulfurovum lithotrophicum* 42BKT^T^

10.1601/nm.8874 42BKT^T^ is a Gram-negative, non-motile, coccoid-to-short-rod-shaped bacterium that is 0.5–1.2 μm in length and 0.4–0.8 μm in width (Fig. 2). The 42BKT^T^ strain is a mesophilic, facultative anaerobe that requires sea salt to grow and can use NH_4_Cl as a nitrogen source. Normal growth occurs at a temperature of 10–40 °C, pH of 5.0–9.0, and salinity of 5–60 g/l [3]. The basic details of its genome sequence are shown in Table 1.

**Table 1:** Classification and general features of *Sulfurovum lithotrophicum* strain 42BKT^T^ [11]

MIGS ID	Property	Term	Evidence code^a^
	Classification	Domain *Bacteria*	TAS [29]
		Phylum *Proteobacteria*	TAS [30]
		Class *Epsilonproteobacteria*	TAS [31]
		Order *Campylobacterales*	TAS [32]
		Family *Helicobacteraceae*	TAS [33]
		Genus *Sulfurovum*	TAS [3]
		Species *Sulfurovum lithotrophicum*	TAS [3]
		Type strain: 42BKT^T^ (CP011308)	TAS [3]
	Gram stain	Negative	TAS [3]
	Cell shape	Coccoid to short rods	TAS [3]
	Motility	None-motile	TAS [3]
	Sporulation	Not reported	NAS
	Temperature range	10–40 °C	TAS [3]
	Optimum temperature	28–30 °C	TAS [3]
	pH range; Optimum	6.5–7.0	TAS [3]
	Carbon source	Sodium bicarbonate	TAS [4]
MIGS-6	Habitat	Deep-sea hydrothermal vent	TAS [3]
MIGS-6.3	Salinity	0.5–6% NaCl (*w*/*v*)	TAS [3]
MIGS-22	Oxygen requirement	Facultatively anaerobic	TAS [3]
MIGS-15	Biotic relationship	Symbiont	TAS [3]
MIGS-14	Pathogenicity	Not reported	NAS
MIGS-4	Geographic location	Okinawa, Japan	TAS [3]
MIGS-5	Sample collection	April 2002	TAS [3]
MIGS-4.1	Latitude	27° 47·38′ N	TAS [3]
MIGS-4.2	Longitude	126° 53·87′ E	TAS [3]
MIGS-4.4	Altitude	−1033 m	TAS [3]

^a^Evidence codes - *TAS* Traceable Author Statement (i.e., a direct report exists in the literature); *NAS* Non-traceable Author Statement (i.e., not directly observed for the living, isolated sample, but based on a generally accepted property for the species or anecdotal evidence). These evidence codes are from the Gene Ontology project [34]

#### Chemotaxonomic data

The major cellular fatty acids that were present in strain 42BKT^T^ included C_16: 1_ (53.7%), C_16: 0_ (31.3%), and C_18: 0_ (15.0%) [3]. It did not contain C_14:0_, C_14:1_, or C_18:1_, whereas 10.1601/nm.25804 Monchim33^T^ contains 7.7, 5.9, and 9.4%, respectively, of these fatty acids [3, 7], and 10.1601/nm.3857 OK 10^T^, another chemolithoautotrophic bacteria, contains 8.4% of C_14:0_ and 9.4% of C_18:1_ [8]. 10.1601/nm.8874 42BKT^T^ can fix CO_2_ via the reductive tricarboxylic acid (TCA) cycle, although the gene encoding phosphoenolpyruvate (PEP) carboxylase is not annotated in its genome. Sulfur or S_2_O_3_^2−^ are oxidized by bacteria of the genus 10.1601/nm.8873; 10.1601/nm.8874 42BKT^T^ can oxidize S^2−^ only using a sulfide-quinone reductase, whereas 10.1601/nm.8873 sp. NBC37–1 oxidizes S^2−^ using a sulfide-quinone reductase or a sulfide dehydrogenase.

## Genome sequencing information

### Genome project history

10.1601/nm.8874 42BKT^T^ was selected for sequencing based on its ability to convert CO_2_ into C_5_H_8_NO_4_^−^, which can be industrially used for CO_2_ bio-mitigation. The draft sequencing and annotation were performed by ChunLab, Inc. (Seoul, Korea). The genome project was deposited in the Genomes OnLine Database [9] under the accession number Gp0118364. The complete genome sequence was also deposited in GenBank [10] under the accession number CP011308. Table 2 contains the details of the project and its association with MIGS version 2.0 compliance [11].

**Table 2:** Project information

MIGS ID	Property	Term
MIGS 31	Finishing quality	Completely finished
MIGS 28	Libraries used	Illumina 300-bp paired-end library, PacBio 20 K library
MIGS 29	Sequencing platforms	Miseq PE 300, PacBio 10 K
MIGS 31.2	Fold coverage	852.21×
MIGS 30	Assemblers	CLC Genomics Workbench v.7.5.1, SMRT Analysis v.2.3
MIGS 32	Gene-calling method	Prodigal 2.6.2
	Locus Tag	YH65
	Genbank ID	CP011308.1
	Genbank Date of Release	08/20/2015
	GOLD ID	Gp0118364
	BIOPROJECT	PRJNA279430
MIGS 13	Source-material identifier	42BKT^T^/ ATCC BAA-797^T^
	Project relevance	CO_2_ fixation

### Growth conditions and genomic DNA preparation

10.1601/nm.8874 42BKT^T^ was grown in a 125-mL serum bottle (Wheaton Industries, Millville, NJ, USA) with 20 mL of MJ basal medium and filled with a CO_2_/N_2_ gas mixture. The bottle was incubated at 29 °C while shaking at 120 rpm (Green Shaker, Vision Scientific Co., Daejeon, Korea) [4]. Genomic DNA was isolated using a QIAmp DNA mini kit (Qiagen, Hilden, Germany), according to the manufacturer’s instructions.

### Genome sequencing and assembly

The genomic library was sequenced using an Illumina MiSeq PE 300 and PacBio 10 K with the Illumina 300-bp paired-end library (Illumina, San Diego, CA, USA) and the PacBio 20 K library (Pacific Biosciences, Menlo Park, CA, USA), respectively. The generated paired-end sequencing reads (total read length: 2217,891 bp) were assembled using the CLC Genomics Workbench version 7.5.1 (CLC Bio, Aarhus, Denmark) and PacBio SMRT Analysis version 2.3 (Pacific Biosciences), resulting in one contig with an average genome coverage of 852.21 × .

### Genome annotation

The genome was annotated using the NCBI Prokaryotic Genome Annotation Pipeline [12], which was designed to annotate bacterial genomes. Genome annotation was performed by predicting protein-coding, rRNA, tRNA, ncRNA, and pseudo genes. Phobius [13] was used to predict signal-peptide genes, and TMHMM Server version 2.0 [14] was used to predict transmembrane helix genes [15, 16]. Protein families [17] were investigated using Pfam 29.0 [18], and GeneMarkS+ [19], which uses alignment data for gene prediction, was used as an annotation tool [20].

## Genome properties

**Fig. 3:**
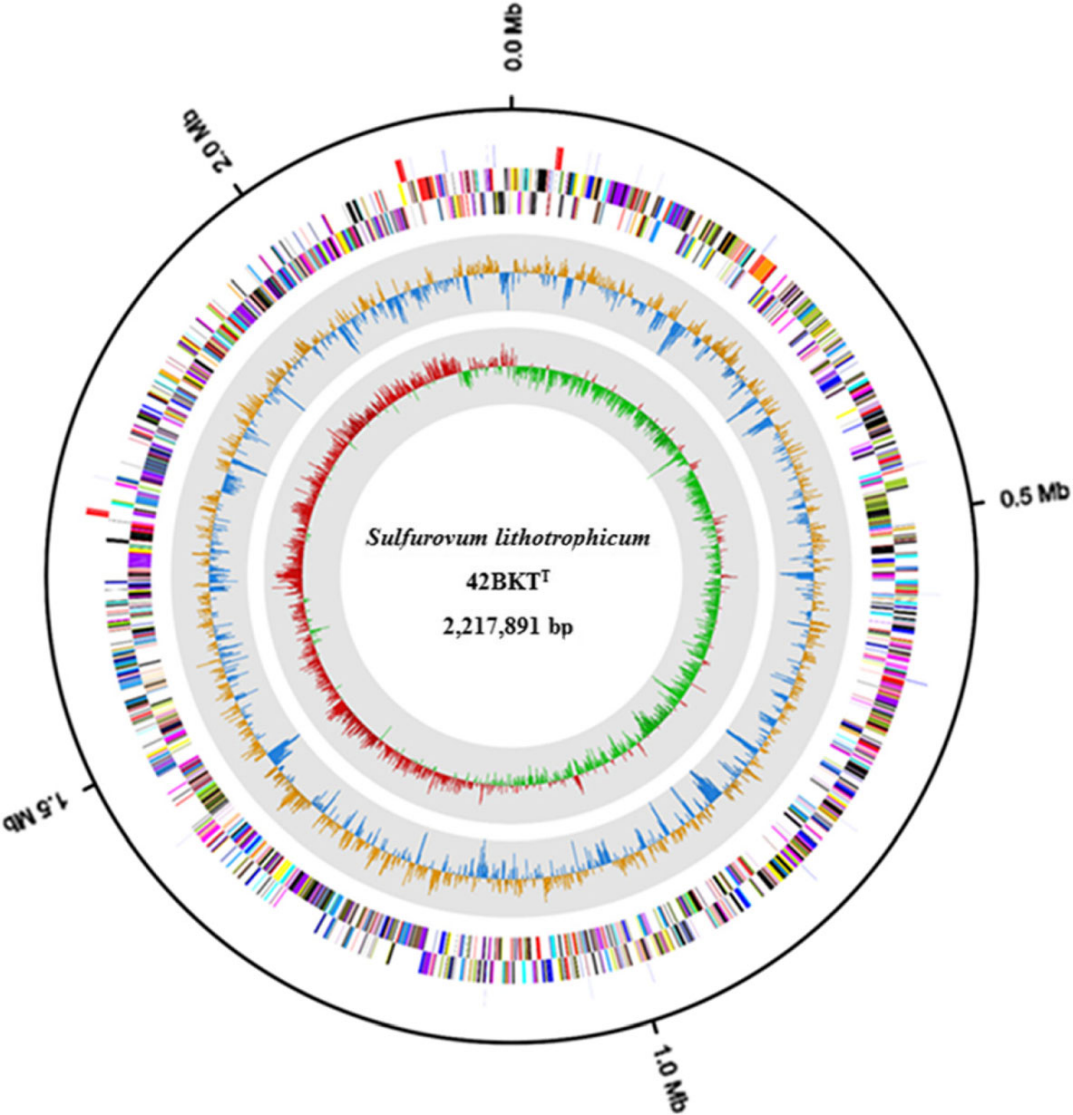
Genome map of *Sulfurovum lithotrophicum* 42BKT^T^. From the outer to the inner circle: RNA regions (rRNA, red; tRNA, lavender), CDS on the reverse strand (colored based on COG categories), CDS on the forward strand (colored based on COG categories), G + C skew (*blue/goldenrod*), and GC ratio (*green*/*red*)

The genome of 10.1601/nm.8874 42BKT^T^ comprised a single circular chromosome of 2217,891 bp with a GC content of 44.26%. Among the 2217 genes predicted, 2146 (96.80%) were protein-coding DNA sequences, 17 of which were pseudogenes. Among the CDSs, 89.66% were grouped into cluster of orthologous group functional categories. The genome contained a CRISPR array and 54 RNA genes, including 44 tRNAs, 9 rRNAs, and one ncRNA. The properties and statistics of the genome are summarized in Fig. 3 and Tables 3 and 4, 5.

**Table 3:** Genome statistics

Attribute	Value	% of total
Genome size (bp)	2217,891	100.00
DNA coding (bp)	2,028,222	91.44
DNA G + C (bp)	981,638	44.26
DNA scaffolds	1	
Total genes	2217	100.00
Protein-coding genes	2146	96.80
RNA genes	54	2.44
Pseudo genes	17	0.77
Genes in internal clusters	NA	NA
Genes with function prediction	1559	70.32
Genes assigned to COGs	1979	89.26
Genes with Pfam domains	1770	79.84
Genes with signal peptides	412	18.58
Genes with transmembrane helices	513	23.14
CRISPR repeats	1

**Table 4:** Number of genes associated with the general COG functional categories

Code	Value	% age^a^	Description
J	138	6.43	Translation, ribosomal structure, and biogenesis
A	0	0.00	RNA processing and modification
K	47	2.19	Transcription
L	94	4.38	Replication, recombination, and repair
B	1	0.05	Chromatin structure and dynamics
D	14	0.65	Cell cycle control, cell division, chromosome partitioning
V	18	0.84	Defense mechanisms
T	88	4.10	Signal-transduction mechanisms
M	144	6.71	Cell wall/membrane/envelope biogenesis
N	6	0.28	Cell motility
U	39	1.82	Intracellular trafficking and secretion
O	95	4.43	Post-translational modification, protein turnover, chaperones
C	138	6.43	Energy production and conversion
G	53	2.47	Carbohydrate transport and metabolism
E	119	5.55	Amino acid transport and metabolism
F	60	2.80	Nucleotide transport and metabolism
H	85	3.96	Coenzyme transport and metabolism
I	43	2.00	Lipid transport and metabolism
P	106	4.94	Inorganic ion transport and metabolism
Q	22	1.03	Secondary metabolites biosynthesis, transport and catabolism
R	143	6.66	General function prediction only
S	526	24.51	Function unknown
-	238	11.09	Not in COGs

^a^Percentage of the total number of protein-coding genes in the genome

**Table 5:** Species in the genus *Sulfurovum*

Species (isolation source)	Genome size (Mb)	Accession no.	CDS	GC (%)	Reference
*Sulfurovum lithotrophicum* 42BKT^T^ (Deep-sea hydrothermal sediment)	2.21	CP011308	2092	44.3	This report
*Sulfurovum* sp. NBC37–1 (Deep-sea hydrothermal vent)	2.56	AP009179	2466	43.8	[6]
*Candidatus* Sulfurovum sediminum AR (Marine sediment)	2.12	AJLE01000000	2114	39.2	[26]

## Insights from the genome sequence

10.1601/nm.8874 42BKT^T^ is a sulfur-oxidizing bacterium that can fix CO_2_ through the reductive TCA cycle. Here, we focused on investigating its abilities for CO_2_ fixation and sulfur oxidation (sox), based on its genome sequence.

So far, six pathways have been associated with CO_2_ fixation: the Calvin-Benson-Bassham or reductive pentose pathway, the reductive TCA cycle or reverse citric acid cycle, the reductive acetyl CoA or Wood-Ljungdahl pathway, the 3-hydroxypropionate pathway or malyl CoA pathway, the 3-hydroxypropionate/4-hydroxy-butyrate cycle, and the dicarboxylate/4-hydroxybutyrate cycle [21, 22]. Similar to the majority of 10.1601/nm.3783, 10.1601/nm.8874 42BKT^T^ can also grow chemoautotrophically through its adenosine triphosphate citrate lyase, 2-oxoglutarate:ferredoxin oxidoreductase, and pyruvate:ferredoxin oxidoreductase via the reductive TCA cycle [23–25]. We annotated these three key enzymes, as well as other relevant enzymes such as malate dehydrogenase, fumarate hydratase, fumarate reductase, isocitrate dehydrogenase, aconitate hydratase, PEP synthase, and PEP carboxylase, in the genome sequence of 42BKT^T^. Notably, 10.1601/nm.8873 sp. NBC37–1 and *Candidatus* Sulfurovum sediminum AR could also assimilate CO_2_ via the reductive TCA cycle [6, 26].

10.1601/nm.8874 42BKT^T^ is known to oxidize or S_2_S O_3_^2−^ via a sox system using SoxB, SoxXA, SoxYZ, and Sox(CD)_2_ periplasmic proteins [27]. These enzymes catalyze the oxidation of S or S_2_O_3_^2−^ using horse cytochrome *c* as the final electron acceptor [28]. Here, we confirmed the presence of SoxA, SoxB, SoxZ, SoxY, and SoxX genes in the 42BKT^T^ genome.

## Conclusions

To the best of our knowledge, this is the first report describing the genome sequence of 10.1601/nm.8874 42BKT^T^, which comprised a circular chromosome of 2217,891 bp (44.26% GC content) with 2217 genes, among which 2146 were CDSs, 17 were pseudogenes, and 54 were RNA genes. 10.1601/nm.8874 42BKT^T^ assimilates CO_2_ via the reductive TCA cycle and oxidizes S or S_2_O_3_^2−^ via the sox system. The details of the genome sequence of this strain could provide potential strategies to enhance the industrial application of such bacteria for CO_2_ bio-mitigation.

## Abbreviations

CDS: Coding DNA sequence
COG: Cluster of orthologous group
PEP: Phosphoenolpyruvate
TCA: Tricarboxylic acid

## References

[1] Nakagawa S, Takai K, Inagaki F, Hirayama H, Nunoura T, Horikoshi K, Sako Y (2005). Distribution, phylogenetic diversity and physiological characteristics of epsilon-*Proteobacteria* in a deep-sea hydrothermal field. Environ Microbiol.

[2] Huber JA, Butterfield DA, Baross JA (2003). Bacterial diversity in a subseafloor habitat following a deep-sea volcanic eruption. FEMS Microbiol Ecol.

[3] Inagaki F, Takai K, Nealson KH, Horikoshi K (2004). *Sulfurovum lithotrophicum* gen. nov., sp. nov., a novel sulfur-oxidizing chemolithoautotroph within the epsilon-*Proteobacteria* isolated from Okinawa trough hydrothermal sediments. Int J Syst Evol Microbiol.

[4] Kwon HS, Lee JH, Kim T, Kim JJ, Jeon P, Lee CH, Ahn IS (2015). Biofixation of a high-concentration of carbon dioxide using a deep-sea bacterium: *Sulfurovum lithotrophicum* 42BKTT. RSC Adv.

[5] NCBI BLAST. https://blast.ncbi.nlm.nih.gov/Blast.cgi. Accessed 17 Jan 2017.

[6] Nakagawa S, Takaki Y, Shimamura S, Reysenbach AL, Takai K, Horikoshi K (2007). Deep-sea vent epsilon-proteobacterial genomes provide insights into emergence of pathogens. Proc Natl Acad Sci U S A.

[7] Mino S, Kudo H, Arai T, Sawabe T, Takai K, Nakagawa S (2014). *Sulfurovum aggregans* sp. nov.,a hydrogen-oxidizing, thiosulfate-reducing chemolithoautotroph within the *Epsilonproteobacteria* isolated from a deep-sea hydrothermal vent chimney, and an emended description of the genus *Sulfurovum*. Int J Syst Evol Microbiol.

[8] Inagaki F, Takai K, Kobayashi H, Nealson KH, Horikoshi K (2003). *Sulfurimonas autotrophica* gen. nov., sp. nov., a novel sulfur-oxidizing epsilon-proteobacterium isolated from hydrothermal sediments in the Mid-Okinawa Trough. Int J Syst Evol Microbiol.

[9] Genomes OnLine Database. https://gold.jgi.doe.gov/. Accessed 17 Jan 2017.

[10] GenBank. https://www.ncbi.nlm.nih.gov/genbank/. Accessed 17 Jan 2017.

[11] Field D, Garrity G, Gray T, Morrison N, Selengut J, Sterk P, Tatusova T, Thomson N, Allen MJ (2008). Angiuoli SV and others. The minimum information about a genome sequence (MIGS) specification. Nat Biotechnol.

[12] NCBI Prokaryotic Genome Annotation Pipeline. https://www.ncbi.nlm.nih.gov/genome/annotation_prok/. Accessed 17 Jan 2017.

[13] Phobius. http://phobius.sbc.su.se/. Accessed 17 Jan 2017.

[14] TMHMM Server version 2.0. http://www.cbs.dtu.dk/services/TMHMM/. Accessed 17 Jan 2017.

[15] Kall L, Krogh A, Sonnhammer EL (2007). Advantages of combined transmembrane topology and signal peptide prediction-the Phobius web server. Nucleic Acids Res.

[16] Krogh A, Larsson B, von Heijne G, Sonnhammer EL (2001). Predicting transmembrane protein topology with a hidden Markov model: application to complete genomes. J Mol Biol.

[17] Bateman A, Birney E, Durbin R, Eddy SR, Howe KL, Sonnhammer ELL (2000). The Pfam protein families database. Nucleic Acids Res.

[18] Pfam 29.0. http://pfam.xfam.org/. Accessed 17 Jan 2017.

[19] GeneMarkS+. http://exon.gatech.edu/Genemark/genemarks.cgi. Accessed 17 Jan 2017.

[20] Besemer J, Lomsadze A, Borodovsky M (2001). GeneMarkS: a self-training method for prediction of gene starts in microbial genomes. Implications for finding sequence motifs in regulatory regions. Nucleic Acids Res.

[21] Saini R, Kapoor R, Kumar R, Siddiqi TO, Kumar A (2011). CO(2) utilizing microbes--a comprehensive review. Biotechnol Adv.

[22] Kanao T, Fukui T, Atomi H, Imanaka T (2001). ATP-citrate lyase from the green sulfur bacterium *Chlorobium limicola* is a heteromeric enzyme composed of two distinct gene products. Eur J Biochem.

[23] Hügler M, Gärtner A, Imhoff JF (2010). Functional genes as markers for sulfur cycling and CO2 fixation in microbial communities of hydrothermal vents of the Logatchev field. FEMS Microbiol Ecol.

[24] Hugler M, Wirsen CO, Fuchs G, Taylor CD, Sievert SM (2005). Evidence for autotrophic CO2 fixation via the reductive tricarboxylic acid cycle by members of the epsilon subdivision of proteobacteria. J Bacteriol.

[25] Takai K, Campbell BJ, Cary SC, Suzuki M, Oida H, Nunoura T, Hirayama H, Nakagawa S, Suzuki Y, Inagaki F (2005). Enzymatic and genetic characterization of carbon and energy metabolisms by deep-sea hydrothermal chemolithoautotrophic isolates of *Epsilonproteobacteria*. Appl Environ Microbiol.

[26] Park SJ, Ghai R, Martin-Cuadrado AB, Rodriguez-Valera F, Jung MY, Kim JG, Rhee SK (2012). Draft genome sequence of the sulfur-oxidizing bacterium “Candidatus Sulfurovum sediminum” AR, which belongs to the *Epsilonproteobacteria*. J Bacteriol.

[27] Friedrich CG, Bardischewsky F, Rother D, Quentmeier A, Fischer J (2005). Prokaryotic sulfur oxidation. Curr Opin Microbiol.

[28] Bardischewsky F, Quentmeier A, Rother D, Hellwig P, Kostka S, Friedrich CG (2005). Sulfur dehydrogenase of *Paracoccus pantotrophus*: the heme-2 domain of the molybdoprotein cytochrome c complex is dispensable for catalytic activity. Biochemistry.

[29] Woese CR, Kandler O, Wheelis ML (1980). Towards a natural system of organisms: proposal for the domains Archaea, Bacteria, and Eucarya. Proc Natl Acad Sci U S A.

[30] Garrity GM, Bell JA, Phylum LT (2005). XIV. Proteobacteria phyl. nov. Bergey’s manual of systematic bacteriology.

[31] Garrity GM, Bell JA, Lilburn T (2005). Class V. *Epsilonproteobacteria* class. nov. Bergey’s manual of systematic bacteriology.

[32] Garrity GM, Bell JA, Lilburn T (2005). Order I. *Campylobacterales* ord. nov. Bergey’s manual of systematic bacteriology.

[33] Garrity GM, Bell JA, Lilburn T (2005). Family II. *Helicobacteraceae* fam. nov. Bergey’s manual of systematic bacteriology.

[34] Ashburner M, Ball CA, Blake JA, Botstein D, Butler H, Cherry JM, Davis AP, Dolinski K, Dwight SS, Eppig JT (2000). Gene ontology: tool for the unification of biology. The gene ontology consortium. Nat Genet.

[35] Saitou N, Nei M (1987). The neighbor-joining method: a new method for reconstructing phylogenetic trees. Mol Biol Evol.

[36] Tamura K, Nei M, Kumar S (2004). Prospects for inferring very large phylogenies by using the neighbor-joining method. Proc Natl Acad Sci U S A.

[37] Felsenstein J (1985). Confidence limits on phylogenies: an approach using the bootstrap. Evolution.

[38] Tamura K, Stecher G, Peterson D, Filipski A, Kumar S (2013). MEGA6: molecular evolutionary genetics analysis version 6.0. Mol Biol Evol.

